# Morphological and phylogenetic evaluation of a new species of *Rhodoveronaea* (Rhamphoriaceae, Rhamphoriales) associated with *Quercusfabrei* (Fagales) in Yunnan Province, China

**DOI:** 10.3897/BDJ.13.e154654

**Published:** 2025-07-15

**Authors:** Yan-Yan Yang, Chitrabhanu S. Bhunjun, Vinodhini Thiyagaraja, Pattana Kakumyan, Darbhe J. Bhat, Fatima Al-Otibi, Tai-Shun Li, Qi Zhao, Kevin D. Hyde

**Affiliations:** 1 State Key Laboratory of Phytochemistry and Natural Medicines, Yunnan Key Laboratory for Fungal Diversity and Green Development, Kunming Institute of Botany, Chinese Academy of Sciences, Kunming 650201, China State Key Laboratory of Phytochemistry and Natural Medicines, Yunnan Key Laboratory for Fungal Diversity and Green Development, Kunming Institute of Botany, Chinese Academy of Sciences Kunming 650201 China; 2 School of Science, Mae Fah Luang University, Chiang Rai 57100, Thailand School of Science, Mae Fah Luang University Chiang Rai 57100 Thailand; 3 Center of Excellence in Fungal Research, Mae Fah Luang University, Chiang Rai 57100, Thailand Center of Excellence in Fungal Research, Mae Fah Luang University Chiang Rai 57100 Thailand; 4 Microbial Products and Innovations Research Group, Mae Fah Luang University, Chiang Rai 57100, Thailand Microbial Products and Innovations Research Group, Mae Fah Luang University Chiang Rai 57100 Thailand; 5 Vishnugupta Vishwavidyapeetam, Ashoke, Gokarna 581326, India Vishnugupta Vishwavidyapeetam, Ashoke Gokarna 581326 India; 6 Department of Botany and Microbiology, College of Science, King Saud University, P.O. Box 2455, Riyadh 11451, Saudi Arabia Department of Botany and Microbiology, College of Science, King Saud University, P.O. Box 2455 Riyadh 11451 Saudi Arabia; 7 Department of Plant Pathology, College of Agriculture, Guizhou University, Guiyang Guizhou 550025, China Department of Plant Pathology, College of Agriculture, Guizhou University Guiyang Guizhou 550025 China

**Keywords:** new species, asexual morph, Fagaceae, phylogeny, taxonomy

## Abstract

**Background:**

During an extensive mycological exploration of Fagales-associated mycoflora within the biodiverse landscapes of Yunnan Province, China, we discovered a new saprotrophic fungal species. Based on its unique morphological characteristics and phylogenetic analyses of combined LSU, ITS, SSU, tef1-α and rpb2 sequences, we established *Rhodoveronaeaquerci* as a new member of the genus *Rhodoveronaea*, which was collected from *Quercusfabrei* colonising terrestrial habitats.

**New information:**

*Rhodoveronaeaquerci* is currently only known from its asexual form, which is a morphologically distinct hyphomycete fungus. The distinguishing characteristics of *R.querci* are straight or slightly flexuous, septate and unbranched conidiophores, terminally, integrated, apically protuberant, recurved conidiogenous cells with conspicuous conidiogenous loci and ellipsoidal to narrowly-obovoid, mostly 1-septate, slightly constricted at the central septum, guttulate, conidia with a flat basal scar. *Rhodoveronaeaquerci* and *R.hainanensis* are highly similar in the size and shape of their conidiophores and conidia, but they can be distinguished by other morphological characteristics and by genetic differences. *Rhodoveronaea* species are widely distributed in Europe and Asia and recent studies revealed several new *Rhodoveronaea* species from China.

## Introduction

*Rhodoveronaea* Arzanlou, W. Gams & Crous was introduced by Arzanlou et al. (2007) with *R.varioseptata* Arzanlou, W. Gams & Crous as the type species. The genus showed a close phylogenetic association with the family Annulatascaceae, based on 28S rDNA sequences. Later, Réblová and Štěpánek (2018) introduced the family Rhamphoriaceae to accommodate *Rhodoveronaea* along with three more genera including, *Rhamphoria* Niessl, *Rhamphoriopsis* Réblová & Gardiennet and *Xylolentia* Réblová. Later, the family was placed in its own order Rhamphoriales that was established, based on multigene phylogenetic analysis and divergence time estimates (Hyde et al. 2021).

The asexual morph of *Rhodoveronaea* is characterised by reddish-brown, straight or flexuose conidiophores with inflated basal cells, terminally integrated conidiogenous cells with crowded, slightly conspicuous conidium-bearing denticles, pale brown, ellipsoidal to obovoidal and septate conidia with a protruding base and a marginal basal frill (Arzanlou et al. 2007, Luo et al. 2019, Yuan et al. 2020, Yang et al. 2023, Chen et al. 2024). The sexual morph is distinguished by immersed ascomata with subglobose to conical venter bearing conical neck, filamentous, septate paraphyses longer than asci, unitunicate, cylindrical asci with long-stipitate and fusiform, septate, hyaline ascospores (Réblová 2009, Luo et al. 2019). To date, *Rhodoveronaeavarioseptata* is the only known sexual morph within the genus. The asexual morph of *R.varioseptata* was initially identified on *Bertiamoriformis* (Tode) De Not. in Germany and the sexual morph was later documented on *Carpinusbetulus* L. in the Czech Republic (now Czechia) (Réblová 2009).

Currently, this genus includes seven species: *Rhodoveronaeaaquatica* Z.L. Luo, K.D. Hyde & Hong, *R.everniae* Crous & Boers, *R.hainanensis* Jian Ma, K.D. Hyde & Y.Z. Lu, *R.hyalina* X.M. Chen & Karun., *R.lignicola* X.M. Chen & Tibpromma, *R.nieuwwulvenica* Crous & Osieck and *R.varioseptata*. Amongst these, four species were collected in China, whereas the remaining were described from the Netherlands and the Czechia (Réblová 2009, Crous et al. 2023, Hyde et al. 2023a, Chen et al. 2024). These taxa have been found on different hosts in terrestrial and freshwater habitats (Arzanlou et al. 2007, Réblová 2009, Luo et al. 2019, Crous et al. 2021, Crous et al. 2023, Hyde et al. 2023a, Yang et al. 2023, Chen et al. 2024). Notably, *Rhodoveronaeaaquatica* and *R.hainanensis* are specifically known from freshwater habitats in China, while the other species have been collected from terrestrial habitats (Luo et al. 2019, Hyde et al. 2023b).

Despite their occurrence in diverse ecological niches, the diversity and ecological roles of *Rhodoveronaea* species within the order Fagales, particularly in the context of the family Fagaceae, remain largely unexplored. Although the ecological significance of Fagaceae, research on the fungal diversity associated with this family remains limited, particularly regarding saprobic fungi and their ecological interactions (Wang et al. 2011, Li et al. 2012, Jiang et al. 2022, Phukhamsakda et al. 2022, Jayawardena et al. 2023, Yang et al. 2023, Hyde et al. 2024).

During a comprehensive survey of microfungi associated with Fagales in Yunnan Province, China, we encountered a previously undocumented species of Rhodoveronaea on decayed wood of *Quercusfabrei* Hance (Thiyagaraja et al. 2024). Based on integrated morphological and multi-locus phylogenetic analyses, this isolate was recognised as a novel taxon. This discovery enriches the taxonomic framework of *Rhodoveronaea* and sheds light on the microfungal diversity within Fagaceae in Yunnan, an area known for its rich biodiversity.

## Materials and methods

### Sample collection, morphological study and isolation

**Figure 1.**
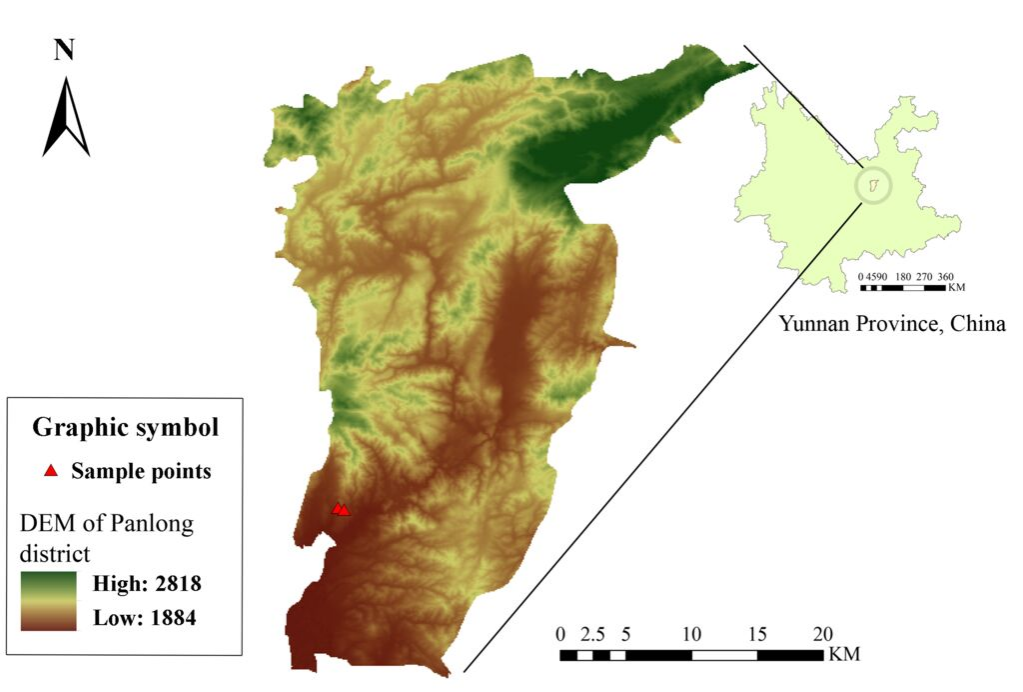
Map of the northern urban area of Kunming City with altitude information.

Dead sticks of *Quercusfabrei* were collected during a survey of Fagales associated microfungi in Kunming City, Yunnan Province, China during the rainy season (October 2021). Kunming, located in a typical subtropical plateau monsoon climate zone, is characterised by mild temperatures throughout the year. The annual average temperature is approximately 15°C and the annual precipitation averages 1000 mm, with most rainfall occurring between May and October. The city’s average altitude is around 1,891 m, contributing to its distinct climate features. The vegetation types in this region mainly include evergreen broad-leaved forests, coniferous forests, shrublands and grasslands, as well as artificial forests and farmland, forming a diverse ecological environment that provides favourable conditions for fungal growth. The two specimens used in this study were collected from the naturally growing Fagaceae forest in Heilongtan Park in the northern area of Kunming City, with loci of 25.143730N, 102.749002E, 25.142479N, 102.753244E, respectively (Fig. 1).

Samples were packed in sealed bags and taken to the laboratory for further observation. Materials were examined with a Nikon SMZ 745T stereomicroscope and the macromorphological characters of the hyphomycetes were photographed using a IMG SC2000C camera. Fruiting bodies were picked up with a needle and placed on a slide with distilled water for observation of microscopic characters (Senanayake et al. 2020). The microscopic characteristics were observed using a Nikon ECLIPSE Ni-U microscope (Model Eclipse Ni-UNikon Corporation Tokyo, Japan) and the structures of conidiophores, conidiogenous cells and conidia were photographed by the charge-coupled camera (IMG SC2000C, Jiangsu Province, China). The photo plates were edited with Adobe Photoshop 2019 and measurements were taken using the Tarosoft (R) Image Frame Work programme. A pure culture was obtained by single spore isolation as described by Senanayake et al. (2020). The germinated spores were transferred to a fresh potato dextrose agar (PDA) plate and grew to 2.5 cm in diameter after being incubated at 25–27°C for two months. The characteristics of the culture were frequently checked. Dry-herbarium specimens and living cultures were deposited in the Kunming Institute of Botany Academia Sinica (HKAS) and Kunming Institute of Botany Culture Collection (KUMCC), respectively. Index Fungorum number and Faces of Fungi number were registered as outlined by Index Fungorum (2024) and Jayasiri et al. (2015). The data are also deposited in the Greater Mekong Subregion database (Chaiwan et al. 2021) and Fungalpedia webpage (Hyde et al. 2023b).

### DNA extraction, PCR amplification and sequencing

Mycelia were scraped from the growing culture and DNA was extracted using a Trelief^TM^ Plant Genomic DNA extraction kit (TsingKe, Beijing, China) following the manufacturer’s instructions. The large subunit ribosomal rRNA (LSU), internal transcribed spacer (ITS) region, small subunit of the nuclear ribosomal DNA (SSU) and translation elongation factor (*tef1-α*) were amplified with primers LR0R/LR5 (Vilgalys and Hester 1990), ITS1F/ITS4 (White et al. 1990, Gardes and Bruns 1993), NS1/NS4 (Rehner and Samuels 1994) and ef1-983F/ef1-2218R (Rehner and Samuels 1994), respectively. The PCR reactions contained 21 μl of 1×PCR Master Mix, 1 μl of each forward and reverse primer and 2 μl of genomic DNA. The following thermo-cycling parameters were used for ITS, LSU, SSU and *tef1-α*: initially, 95℃ for 3 min, followed by 35 cycles of denaturation at 95℃ for 15 s, annealing at 54℃ for 15 s, elongation at 72℃ for 20 s and final extension at 72℃ for 5 min. The amplified PCR products were sequenced in Tsingke (Kunming, China). Generated sequences were deposited in GenBank,and accession numbers were obtained.

### Sequence alignment and phylogenetic analyses

The newly-generated forward and reverse sequences were assembled using Sequencing Project Management (SeqMan) (Clewley 1995). The combined sequences were subjected to BLASTn search at the National Center for Biotechnology Information (NCBI) to identify closely-related taxa. Related species were downloaded from GenBank following recent publications (Table 1) (Chen et al. 2024). The sequence of individual gene was aligned using MAFFT v. 7 (Katoh et al. 2019) and the aligned sequences were trimmed using TrimAl v. 1.2 with default settings (Castresana 2000, Talavera and Castresana 2007). Sequences of all genes were combined using Sequence Matrix v. 1.7.8 (Vaidya et al. 2011). Phylogenetic analyses were performed following Dissanayake et al. (2020). Maximum Likelihood (ML) analysis of the combined multi-gene dataset was performed using RAxML-HPC2 on XSEDE v. 8.2.12 in the CIPRES Science Gateway v. 3.3 (https://www.phylo.org) with 1000 bootstrap iterations and GTR+I+G substitution model. For Bayesian Inference (BI) analysis, MrModelTest v. 2.2 (Nylander 2004) was used in the Akaike Information Criterion (AIC) to obtain the best-fit evolutionary model of each genetic marker. The posterior probabilities (PP) were performed by Markov Chain Monte Carlo sampling (MCMC) in MrBayes v. 3.1.2. Six simultaneous Markov chains were run for 1,000,000 generations and trees were sampled every 100^th^ generation. The first 25% of trees were discarded in the burn-in phase, while the remaining 75% were used to calculate PP in the majority rule consensus tree (Hua et al. 2006). The generated tree was viewed in FigTree v. 1.4.0 (http://tree.bio.ed.ac.uk/software/figtree) and saved for editing in Adobe Illustrator 2020 (Adobe Inc., United States).

## Taxon treatments

### Rhodoveronaea querci

Y. Y. Yang & Q. Zhao sp. nov.

5F165AAE-CB98-50F6-8148-5EC5E8622BDF

859526

#### Materials

- **Type status:** Holotype. **Occurrence:** catalogNumber: HKAS 145561; occurrenceRemarks: on dead sticks of *Quercusfabrei*; recordNumber: YYY79; recordedBy: Y. Y. Yang; occurrenceID: E2970614-75ED-5C09-9C9D-0208B4B658C1; **Taxon:** kingdom: Fungi; phylum: Ascomycota; class: Sordariomycetes; order: Rhamphoriales; family: Rhamphoriaceae; genus: Rhodoveronaea; **Location:** country: China; stateProvince: Yunnan; county: Kunming; **Identification:** identifiedBy: Y. Y. Yang; **Event:** year: 2021; month: October; day: 6; habitat: terrestrial; **Record Level:** institutionCode: Herbarium of Kunming Institute of Botany, Academia Sinica (KUN-HKAS)

#### Description

*Saprobic* on dead sticks of *Quercusfabrei*. **Sexual morph**: Undetermined. **Asexual morph**: Hyphomycetous. *Colonies* on natural substrate superficial, effuse, hairy, scattered or aggregated, with yellowish-brown conidial masses at the apex. *Mycelium* mostly immersed, partly superficial, composed of filamentous, septate, branched, brown to pale brown hyphae. *Conidiophores* 85–129 µm × 3–4.5 µm (x̅ = 101 × 3.5 µm, n = 15), macronematous, mononematous, erect, simple, cylindrical, straight or slightly flexuous, solitary, septate, unbranched, dark-brown, pale brown towards fertile apex, slightly rough-walled, often percurrently proliferating from cut ends. *Conidiogenous cells* polyblastic, integrated, indeterminate, terminal, sympodial, pale brown, cylindrical below, apically protuberant, recurved with conspicuous conidiogenous loci. *Conidia* 8–14 µm × 3–5.5 µm (x̅ = 11 × 4 µm, n = 30), acropleurogenous, ellipsoidal to narrowly obovoid, apically rounded, with a flat basal scar, pale brown, 1–2-septate, mostly 1-septate, slightly constricted at the central septum, guttulate, smooth-walled. *Conidial secession* schizolytic.

Culture characteristics: Conidia germinating on PDA within 24 h. Colonies slow growing on PDA media, reaching 2.5 cm after 2 months at 18℃, offwhite, irregularly-shaped with obvious concentric rings, raised, pleated, with irregular edges, with sparse mycelia; reverse greige, with white mycelial ring.

#### Diagnosis

Similar to *R.hainanensis* in conidial size and morphology, but differing in the thickness of the conidiophore wall, as well as the septation and guttulation of the conidia.

#### Etymology

The species epithet "*querci*" refers to the host genus.

#### Notes

**Figure 2.**
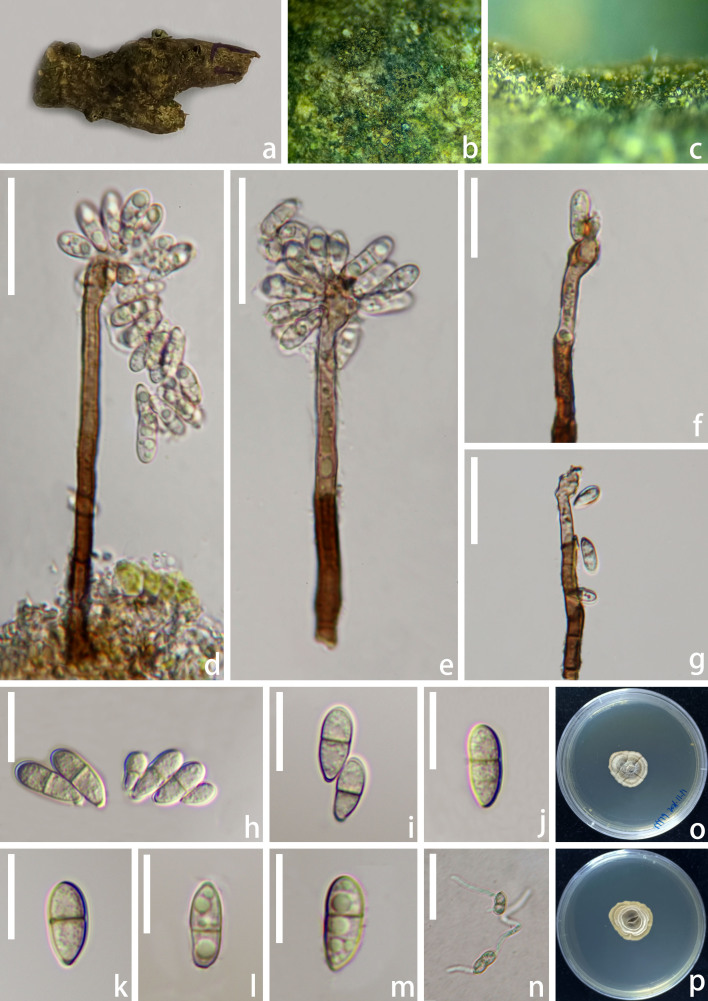
*Rhodoveronaeaquerci* (HKAS 145561, holotype). **a** Host substrate; **b, c** Colonies on the substrate; **d, e** Conidiophores and conidiogenous cells bearing conidia; **f, g** Conidiogenous cells bearing conidia. Note the percurrent proliferation of conidiophores from cut-ends in e-g; **h-m** Conidia; **n** Germinated conidium; **o, p** Upper and lower view of culture on PDA media after incubation for 60 days. Scale bars: **d–e, g, n** = 30 μm, **f** = 20 μm, **h–m** = 10 μm.

In the phylogenetic analyses, our species formed a sister clade to *R.aquatica*, *R.hainanensis* and *R.nieuwwulvenica* (Fig. 2). Pairwise comparisons revealed that *R.querci* differs from *R.aquatica* by 23/506 bp (4.5%) in the ITS sequences, 6/695 bp (0.8%) in the LSU sequences, 0/712 bp (0%) in the SSU sequences and 36/776 bp (4.6%) in the *tef1-α* sequences. Furthermore, *R.querci* differs from *R.hainanensis* by 18/460 bp (3.9%) in the ITS sequences and 12/721 bp (1.7%) in the LSU sequences. Additionally, *R.querci* differs from *R.nieuwwulvenica* by 23/490 bp (4.7%) in the ITS sequences, 9/721 bp (1.2%) in the LSU sequences and 38/744 bp (5.1%) in the *tef1-α* sequences. Morphologically (Table 2), *Rhodoveronaeaquerci* resembles *R.aquatica*, *R.hainanensis* and *R.nieuwwulvenica* by having cylindrical, septate, straight or flexuous, red-brown conidiophores, as well as ellipsoid to obovoid, pale brown conidia (Luo et al. 2019, Hyde et al. 2023a, Crous et al. 2023). However, *R.querci* can be distinguished from *R.aquatica* by its shorter conidiophores (85–129 µm × 3–4.5 µm vs. 182–310 × 9–13 µm) and smaller conidia (8–14 µm × 3–5.5 µm vs. 23–27 × 9–11 µm) (Luo et al. 2019). In contrast to *R.hainanensis*, *R.querci* possesses integrated conidiogenous cells and conidia with guttulate cells and fewer septa（1–2-septate vs. 1–3-septate) (Hyde et al. 2023a). Furthermore, the difference between *R.querci* and *R.nieuwwulvenica* is that *R.nieuwwulvenica* has flexuous, shorter conidiophores (20–60 × 4–5 µm vs. 85–129 µm × 3–4.5 µm) and more septate conidia (3-septate vs. 1–2-septate) (Crous et al. 2023). We therefore introduce this strain as a new species following the guidelines of Chethana et al. (2021).

## Analysis

**Figure 3.**
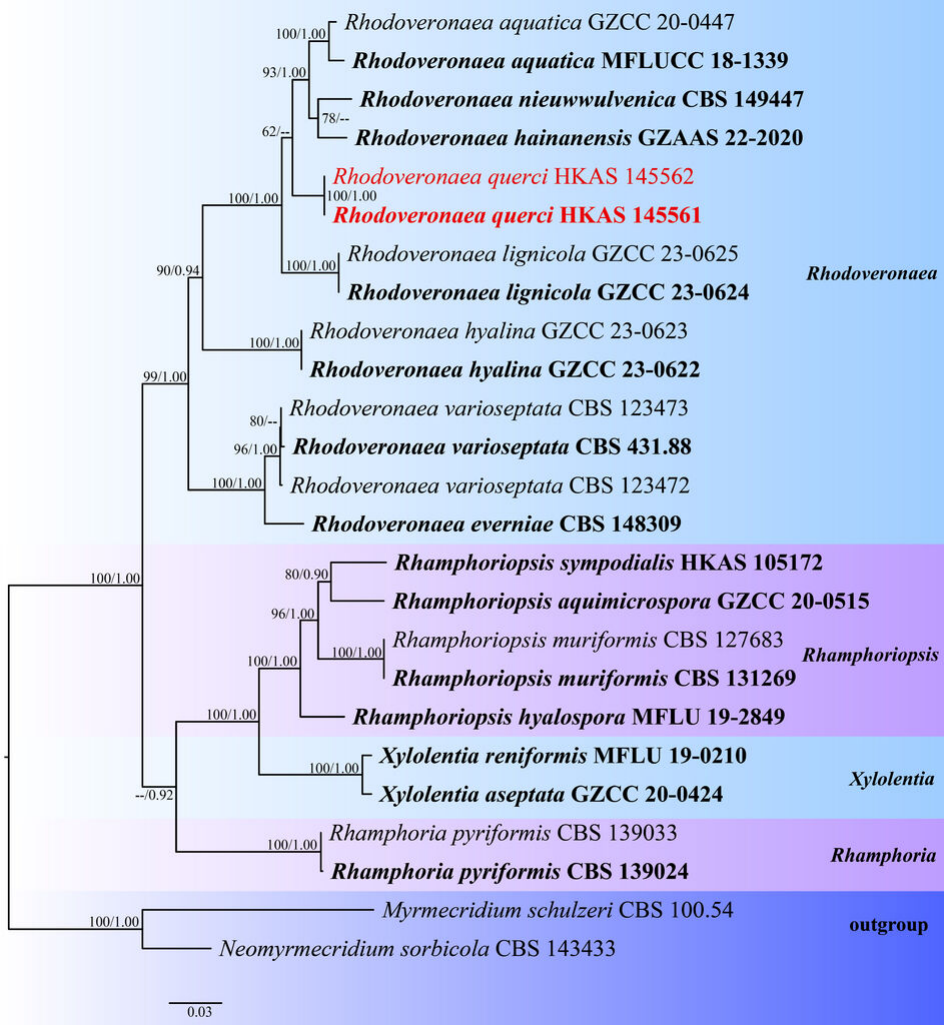
RAxML tree, based on combined LSU, ITS, SSU, *tef1-α* and *rpb2* dataset analyses. Bootstrap support values for Maximum Likelihood (ML, first value) equal to or greater than 60% and Bayesian posterior probabilities (BPP, second value) equal to or greater than 0.90 are given at the nodes. Ex-type strains are in bold; the newly-generated isolates are in red.

Phylogenetic trees were constructed using 25 isolates, including two newly-generated sequences (Table 1). The concatenated dataset consisted of 4614 characters including gaps (LSU: 1-870 bp, ITS: 871-1403 bp, SSU 1404-2708 bp, *tef1-α*: 2709-3605 bp and *rpb2*: 3606-4614 bp). *Myrmecridiumschulzeri* (CBS 100.54) and *Neomyrmecridiumsorbicola* (CBS 143433) were used as outgroup taxa following Chen et al. (2024). The final optimised likelihood value of the ML tree with the highest score was -16176.373461. The alignment had 1073 distinct alignment patterns, with 35.50% of undetermined characters and gaps. The parameters for the GTR+I+G model of combined LSU, ITS, SSU, *tef1-α* and *rpb2* were as follows: estimated base frequencies A = 0.251898, C = 0.251105, G = 0.274700, T = 0.222296; substitution rates AC = 1.481862, AG = 2.986319, AT = 1.330144, CG = 1.336388, CT = 9.252404, GT = 1.000000; and gamma distribution shape parameter α = 0.636081. Phylogenetic analysis showed that *Rhodoveronaeaquerci* clustered with the other three *Rhodoveronaea* species and formed a basal lineage with 62% ML support (Fig. 3).

## Discussion

In this study, we introduce a new saprobic species of *Rhodoveronaea* associated with Fagales, based on phylogenetic studies of combined LSU, ITS, SSU, *tef1-α* and *rpb2* datasets, as well as morphological details. Previous studies by Arzanlou et al. (2007) employed LSU and ITS sequences to classify *Rhodoveronaea*. More recently, multi-gene datasets, such as LSU-ITS-*tef1-α*-*rpb2* and LSU-ITS-SSU-*tef1-α*-*rpb2*, have enhanced the phylogenetic resolution of the genus (*Crous et al. 2023, Hyde et al. 2023a*). However, some species still lack gene fragments, as in *R.everniae* and *R.hainanensis*, which only have data on ITS and LSU sequences (Crous et al. 2021, Hyde et al. 2023a). In future studies, it will be necessary to collect and sequence more gene regions of *Rhodoveronaea* samples.

In our phylogenetic trees, *Rhodoveronaeaquerci* formed a distinct clade sister to *R.aquatica*, *R.nieuwwulvenica* and *R.hainanensis* with low bootstrap support (62 ML/0.68 PP). This relationship was consistent across repeated phylogenetic analyses, with significant differences observed in their nucleotide comparisons (Table 3). Morphologically, *Rhodoveronaeaquerci* is similar to *R.hainanensis* and *R.aquatica* in having unbranched, red-brown conidiophores with septate, ellipsoidal to narrowly obovoid, pale brown and guttulate conidia, but differed from the latter by having flexuous conidiogenous cells with protuberant, slightly recurved apices and conidia with more septa (1–2-septate vs. 1–3-septate) (Luo et al. 2019). *Rhodoveronaeaquerci* shares similarities with *R.nieuwwulvenica* in having terminal, integrated conidiogenous cells and comparably similar-sized guttulate conidia (8–14 × 3–5.5 µm vs. 8–14 × 3–4.5 µm). However, it can be distinguished by its larger conidiophores (85–129 × 3–4.5 µm vs. 20–60 × 4–5 µm) and fewer septa in conidia (1–2-septate vs. 3-septate) (Crous et al. 2023).

*Rhodoveronaea* species have been reported on different hosts, such as *Bertiamoriformis* (fungal substrate), *Everniaprunastri* (L.) Ach. (Parmeliaceae), *Carpinusbetulus* (Betulaceae) and Bambusoideae (Arzanlou et al. 2007, Réblová 2009, Crous et al. 2021, Crous et al. 2023). In this study, *Rhodoveronaea* was reported for the first time on *Quercusfabrei*. The species of *Rhodoveronaea* are widely distributed in Asia and Europe and most species are collected in the temperate region, with only a few from tropical and subtropical regions (Arzanlou et al. 2007, Réblová 2009, Luo et al. 2019, Crous et al. 2021, Crous et al. 2023, Hyde et al. 2023a). Due to the limited ecological and distributional data available, it remains uncertain whether host-specificity and climatic conditions influence the growth of *Rhodoveronaea* species. Collecting *Rhodoveronaea* species poses significant challenges due to their minute, hyphomycetous morphology and inconspicuous growth habits, which often render them overlooked in field studies. Currently, five species have been reported in China and two species have been found on Fagales. *Rhodoveronaeavarioseptata* (CBS 123472) and *R.querci* were isolated as saprobes from *Carpinusbetulus* (Betulaceae, Fagales) and *Quercusfabrei* (Fagaceae, Fagales), respectively (Réblová 2009, Luo et al. 2019, Hyde et al. 2023a, Chen et al. 2024). The differences between *R.varioseptata* and *R.querci* are the conidiogenous cells of *R.varioseptata* have up to 15 sympodially produced denticles producing holoblastic conidia holoblastically and the conidia are slightly larger than the latter (Réblová 2009). This study expands our understanding of the diversity and host associations of *Rhodoveronaea*, highlighting the potential for discovering new fungal species on Fagales plants, particularly in understudied regions and habitats. Further investigations may provide insights into the ecological roles and evolutionary history of these fungi.

## Supplementary Material

XML Treatment for Rhodoveronaea querci

## Figures and Tables

**Table 1.** GenBank accession numbers of the taxa used in the phylogenetic analyses in this study.

**Taxa**	**Strain/Voucher**	**GenBank Accession Numbers**					**References**
		**LSU**	**ITS**	**SSU**	***tef1-α***	***rpb2***	
*Myrmecridiumschulzeri*	CBS 100.54	EU041826	EU041769	NA	NA	NA	Arzanlou et al. (2007)
*Neomyrmecridiumsorbicola*	CBS 143433	MH107948	MH107901	NA	NA	NA	Crous et al. (2018)
*Rhamphoriaaquimicrospora*	GZCC 20-0515^T^	OP377911	OP377812	OP377996	OP472992	OP473085	Yang et al. (2023)
*R.hyalospora*	MFLU 19-2849^T^	MN846342	MN846344	NA	NA	NA	Lin et al. (2023)
*R.muriformis*	CBS 131269^T^	MG600396	NA	MG600404	NA	MG600399	Réblová and Štěpánek (2018)
*R.muriformis*	CBS 127683	MG600395	MG600389	MG600403	NA	MG600400	Réblová and Štěpánek (2018)
*R.pyriformis*	CBS 139024^T^	MG600397	MG600392	MG600405	NA	MG600401	Réblová et al. (2016)
*R.pyriformis*	CBS 139033	KT991665	KT991677	MG600406	NA	KT991656	Réblová et al. (2016)
*R.sympodialis*	HKAS 105172^T^	MT079191	MT079187	NA	NA	NA	Hyde et al. (2020)
*Rhodoveronaeaaquatica*	MFLUCC 18-1339^T^	MK849785	MK828641	MK828310	MN194046	NA	Luo et al. (2019)
*R.aquatica*	GZCC 20-0447	OP377947	OP377862	OP378027	OP473041	OP473107	Yang et al. (2023)
*R.everniae*	CBS 148309T	OK663776	OK664737	NA	NA	OK651172	Crous et al. (2021)
*R.hainanensis*	GZAAS 22-2020^T^	OP748932	OP748935	NA	NA	NA	Hyde et al. (2023a)
*R.hyalina*	GZCC 23-0622^T^	PP102207	PP102206	PP102214	PP259403	PP259399	Chen et al. (2024)
*R.hyalina*	GZCC 23-0623	PP102208	PP102211	PP102215	PP259404	PP259400	Chen et al. (2024)
*R.lignicola*	GZCC 23-0624^T^	PP102209	PP102212	PP102216	PP259405	PP259401	Chen et al. (2024)
*R.lignicola*	GZCC 23-0625	PP102210	PP102213	PP102217	PP259406	PP259402	Chen et al. (2024)
*R.nieuwwulvenica*	CBS 149447^T^	OQ629048	OQ628466	NA	OQ627955	OQ627935	Crous et al. (2023)
***R.querci***	**HKAS 145561^T^**	**PV097144**	**PQ932528**	**PV097142**	**PV164602**	**NA**	**This study**
***R.querci***	**HKAS 145562**	**PV097145**	**PQ932529**	**PV097143**	**PV164603**	**NA**	**This study**
*R.varioseptata*	CBS 431.88^T^	EU041870	EU041813	NA	NA	NA	Arzanlou et al. (2007)
*R.varioseptata*	CBS 123472	FJ617559	MG600393	MG600408	NA	JX066701	Arzanlou et al. (2007)
*R.varioseptata*	CBS 123473	FJ617560	KT991676	JX066710	NA	JX066700	Arzanlou et al. (2007)
*Xylolentiaaseptata*	GZCC 20-0424^T^	OP377944	OP377859	OP378024	OP473038	OP473104	Yang et al. (2023)
*X.reniformis*	MFLU 19-0210^T^	MK547648	MK547646	NA	NA	NA	Yuan et al. (2020)

**Table 2.** Synopsis table of prominent morphological and culture characteristics for Fitzroyomyces species.

Species Name	Conidiophores	Conidiogenous cells	Conidia	Culture	References
Rhodoveronaeaaquatica	182–310 × 9–13 µm, cylindrical, straight or flexuose, thick-walled, septate, red-brown, paler at apex.	Polyblastic, terminally integrated, sympodial, smooth.	23–27 × 9–11 µm, acropleurogenous, ellipsoid to obovoid, apically rounded, with a flat basal scar, 1–3-septate, pale brown.	No	Luo et al. (2019)
R.everniae	25–100 × 3–4 μm, subcylindrical, straight to geniculate-sinuous, branched or not, thick-walled, warty, 1–6-septate, red-brown.	15–40 × 3–4 μm, integrated, terminal and intercalary, smooth to warty, forming a rachis of minute pimple-like subdenticulate loci.	(6.5–)9–11(– 12) × (3–)4(–4.5) μm, ellipsoid to obovoid, with a protruding hilum and minute marginal frill, 0–3-septate, pale brown.	Colonies erumpent, spreading, with moderate aerial mycelium and smooth, lobate margin.	Crous et al. (2021)
R.hainanensis	58–172 μm × 5.5–10.5 μm, cylindrical, straight or flexuose, thick-walled, septate, red-brown, paler at apex.	Polyblastic, terminally integrated, sympodial, sometimes branched, smooth.	10–15 μm × 4–5.5 μm, acropleurogenous, ellipsoid to obovoid, apically rounded, with a flat basal scar, 1–3-septate, pale brown.	Colonies circular, flat, red brown at the entire margin, white in the centre, reverse-side red-brown.	Hyde et al. (2023a)
R.hyalina	95–165 × 4.5–8 µm, cylindrical, erect or geniculate-sinuous, smooth-walled, septate, unbranched, red-brown, paler towards the apex.	Polyblastic, integrated, terminal, determinate, sympodial.	14–18 × 4.5–5.5 µm, acropleurogenous, ellipsoidal to narrowly obovoid, apically rounded with a flat basal scar, aseptate, hyaline to subhyaline, guttulate.	Colonies circular, with an umbonate surface, edge regular, obvious rings, rings dark brown, the rest of the grey-brown, reverse black, with inconspicuous circles.	Chen et al. (2024)
R.lignicola	75–125 × 3.5–7.5 µm, cylindrical, straight or slightly flexuous, slightly rough-walled, septate, unbranched, dark brown, paler towards the apex.	Polyblastic, integrated, terminal, determinate, sympodial.	9–13.5 × 4–6 µm, acropleurogenous, ellipsoidal to narrowly obovoid, conical at apex with a flat basal scar, 1–3-septate when mature, hyaline to pale yellowish, guttulate.	Colonies circular white to greyish-white, with an umbonate and irregular edge, reverse light yellow, with inconspicuous circles	Chen et al. (2024)
R.nieuwwulvenica	20–60 × 4–5 µm, subcylindrical, flexuous, thick-walled, 2–3-septate, red-brown.	20–50 × 4–5 µm, integrated, terminal, finely verruculose, forming a rachis with subdenticulate loci.	(8–)11–13(–14) × (3–)4(–4.5) µm, solitary, subcylindrical to narrowly fusoid-ellipsoid, apex subobtuse, base bluntly rounded, 3-septate, medium brown, guttulate.	Colonies erumpent, folded, with sparse aerial mycelium and smooth, lobate margin.	Crous et al. (2023)
R.querci	85–129 µm × 3–4.5 µm, cylindrical, straight or slightly flexuous, slightly rough-walled, septate, unbranched, dark-brown, pale brown towards fertile apex, often percurrently proliferating from cut ends.	Polyblastic, integrated, indeterminate, terminal, sympodial, apically protuberant, recurved with conspicuous conidiogenous loci.	8–14 µm × 3–5.5 µm, acropleurogenous, ellipsoidal to narrowly obovoid, apically rounded, with a flat basal scar, 1–2-septate, pale brown, guttulate.	Colonies off-white with obvious concentric rings, raised, pleated, with irregular edges, with sparse mycelia, reverse greige, with white mycelial ring.	This study
R.varioseptata	80−93 × 4–5 μm, cylindrical, straight or flexuous, brown to red-brown.	Polyblastic, integrated, sympodial, bearing up to 15 sympodially produced denticles.	14–16 × 5−6.5 μm, ellipsoidal to obovoidal, apically rounded, with a flat basal scar and marginal frill, 0−3(−4)-septate, pale brown.	Colonies surface olivaceous-grey to olivaceous-pale brown with a pale grey zone at the margin, aerial mycelium dense, whitish to pale grey, margin entire， reverse olivaceous-black.	Réblová (2009)

**Table 3.** Base differences in *Rhodoveronaeaquerci* compared to other species. “NA”: no data available in GenBank.

**Species**	**Strain**	**ITS**	**LSU**	**SSU**	***tef1-α***
*R.aquatica*	MFLUCC 18-1339	23/506 bp (4.5%)	6/695 bp (0.8%)	0/712 bp (0%)	36/776 bp (4.6%)
*R.hainanensis*	GZAAS 22-2020	18/460 bp (3.9%)	12/721 bp (1.7%)	NA	NA
*R.nieuwwulvenica*	CBS 149447	23/490 bp (4.7%)	9/721 bp (1.2%)	NA	38/744 bp (5.1%)
